# Targeting the Protective Arm of the Renin-Angiotensin System to Reduce Systemic Lupus Erythematosus Related Pathologies in MRL*-lpr* Mice

**DOI:** 10.3389/fimmu.2020.01572

**Published:** 2020-07-23

**Authors:** Maira Soto, Nicole Delatorre, Chelsie Hurst, Kathleen E. Rodgers

**Affiliations:** Pharmacology Department, Center for Innovation in Brain Science, College of Medicine, University of Arizona, Tucson, AZ, United States

**Keywords:** angiotensin, SLE, Mas, ACE-I, ARB, immunomodulation

## Abstract

Patients with Systemic Lupus Erythematosus (SLE) suffer from a chronic inflammatory autoimmune disease that results from the body's immune system targeting healthy tissues which causes damage to various organ systems. Patients with lupus are still in need of effective therapies to treat this complex, multi-system disease. Because polymorphisms in ACE are associated with the activity of SLE and lupus nephritis and based on well-documented renal-protective effects of Renin Angiotensin System (RAS)-modifying therapies, ACE-I are now widely used in patients with SLE with significant efficacy. Our research explores alternate ways of modifying the RAS as a potential for systemic therapeutic benefit in the MRL*-lpr* mouse model of SLE. These therapeutics include; angiotensin (1-7) [A(1-7)], Nor-Leu-3 Angiotensin (1-7) (NorLeu), Losartan (ARB), and Lisinopril (ACE-I). Daily systemic treatment with all of these RAS-modifying therapies significantly reduced the onset and intensity in rash formation and swelling of the paw. Further, histology showed a corresponding decrease in hyperkeratosis and acanthosis in skin sections. Important immunological parameters such as decreased circulating anti-dsDNA antibodies, lymph node size, and T cell activation were observed. As expected, the development of glomerular pathologies was also attenuated by RAS-modifying therapy. Improved number and health of mesenchymal stem cells (MSCs), as well as reduction in oxidative stress and inflammation may be contributing to the reduction in SLE pathologies. Several studies have already characterized the protective role of ACE-I and ARBs in mouse models of SLE, here we focus on the protective arm of RAS. A(1-7) in particular demonstrates several protective effects that go beyond those seen with ACE-Is and ARBs; an important finding considering that ACE-Is and ARBs are teratogenic and can cause hypotension in this population. These results offer a foundation for further pharmaceutical development of RAS-modifying therapies, that target the protective arm, as novel SLE therapeutics that do not rely on suppressing the immune system.

## Introduction

Disease incidence for Systemic Lupus Erythematosus (SLE) has increased over the last 50 years (1, 2). Unfortunately, the development of new therapeutics has not kept pace as only one new medication, belimumab, has been approved over that same time frame (3). As of 2019, several new therapies have shown some success, but are yet to be FDA approved (4). For now, steroids continue to be a mainstay in the treatment of SLE despite their well-documented organ damaging effects. The use of steroids is supplemented with a number of repurposed drugs including anti-malarial drugs, immunosuppressive chemotherapies, and other immunosuppressive agents (5, 6). Current therapeutic pipelines are almost exclusively immunosuppressive therapies that have the potential for several side effects (7, 8). Despite this array of treatments, patients with lupus are still in need of effective therapies to treat this complex, multi-system disease especially those targeting the most lethal manifestations of lupus: cardiac and renal (9–13).

The Renin Angiotensin System (RAS) is widely known for its role in blood pressure regulation. Angiotensin II (A-II), generated by renin and angiotensin-converting enzyme (ACE). ACE2 can then further degrade A-II to angiotensin (1-7) [A(1-7)]. Both A-II and A(1-7) are bioactive peptides that affect various systems. A-II acts through the angiotensin type I (AT1) receptor to mediate such functions as vasoconstriction, increase in insulin resistance, oxidative stress (OS), chronic inflammation, hypertension, and end organ failure (14–17). Because of its central role in hypertension, various anti-hypertensives drugs were developed that target the A-II/AT1 receptor axis; ACE inhibitors (ACE-Is) and Angiotensin Receptor Blockers (ARBs). In contrast to the pathological responses observed by chronic activation of the AT1 arm of the RAS, activation of the protective arm occurs after A(1-7) initiates signaling through Mas or Angiotensin type 2 (AT2) receptor (18, 19). A(1-7) causes vasodilation and decreases OS, fibrosis and inflammation (20, 21).

Polymorphisms in ACE are associated with the activity of SLE (22) and lupus nephritis (23, 24). In some studies, these polymorphisms were thought to increase ACE concentrations and, in turn, increase the incidence of SLE (25). Based on these results and the well-documented renal-protective effects of RAS-modifying therapies, ACE-I were tested as therapy for SLE. In the longitudinal LUMINA study, ACE-I use delayed renal pathologies and overall disease activity in SLE patients (26). Additionally, animal studies have also shown protective effects of ACE-Is in SLE mouse models beyond renal actions such as reduction of fibrosis (27–30).

While the benefit of inhibiting the classical arm of RAS for reno-protection is well-recognized, focus has shifted to the protective arm of RAS. Two potential therapeutic activators of Mas are available; A(1-7) and NorLeu^3^-A(1-7) [NorLeu] a peptide analog of A(1-7). Our studies here show that Mas agonists reduce pathologies and mitigate immune changes in MLR*-lpr* mice to the same levels as ACE-Is/ARBs or better. Mas agonists have the potential to provide alternatives to non-hypertensive patients and those who are starting families, as ACE-Is and ARBs are known to be teratogenic. More importantly, they provide an alternative to immunosuppressive therapy.

## Materials and Methods

### Animal Models

The MRL/MpJ-Faslpr/J (MRL*-lpr*) mouse strain was used as a model for SLE and MRL/MpJ mice served as controls. These mice were treated by daily subcutaneous injections with one of the following: a MRL/MpJ group treated with saline; MRL*-lpr* treated with either saline, 0.5 mg/kg of A(1-7), 0.5 mg/kg NorLeu, 10 mg/kg lisinopril or 10 mg/kg of losartan by once daily subcutaneous injections of treatment starting at 8 weeks of age for 6 weeks. The doses for A(1-7) and NorLeu were chosen from previous studies where 0.5 mg/kg were sufficient to see changes and there was no added benefit from higher doses (31, 32); for lisinopril and losartan we chose doses that have worked for previous studies and are in the range of other doses previously used in SLE mouse models (29, 30). Throughout the study, the development of face lesions/rash, weight, and proteinuria were monitored. Paw edema/inflammation was measured at the end of the study. Mice were anesthetized by isoflurane and blood harvested via cardiac puncture. After euthanasia, the kidney, facial tissues from the snout region, axillary and inguinal lymph nodes, and the spleen were collected. All animal studies have been reviewed and approved by both the University of Arizona and University of Southern California Institutional Animal Care and Use Committees (IACUC).

### Phenotypic Characterization

Facial scoring was completed weekly. Scoring was done according to predetermined criteria: 0, not noticeable rash; 1, little redness, no hair loss or inflammation; 2, minimal rash, little hair loss or inflammation; 3, moderate rash, increased hair loss, light inflammation; 4, pronounced rash, near total hair loss, and obvious inflammation; 5, Rash is traveling up the face; 6, obvious wound above nose. To measure edema/inflammation of the joint, the thickness of the right hind paw was measured after 37 days of treatment using a caliper. The measurement was taken in the middle where the paw is thickest.

### Histological Analysis

Paraffin-embedded kidney, spleen, and facial tissue sections cut at 6 μm and stained with hematoxylin-eosin (H&E). The whole length of the facial tissue, focusing on the top most layers, were photographed at x20 magnification. Hyperkeratosis was measured by dividing the area of the stratum corneum by the length of the tissue. Acanthosis was measured by dividing the area of the stratum Basale/stratum spinosum by the length of the tissue. Skin sections were also stained with Masson's Trichrome Stain and blinded sections were scored. The Singer method was used to score the collagen architecture: 3, normal; 2, abnormal collagen in the papillary dermis; 1, abnormal collagen in the upper reticular dermis only; 0, abnormal collagen in the upper and lower half of the reticular dermis. The whole spleen section was photographed at an x20 magnification. The area of the follicles was measured using the standard software package for the Echo Revolve (San Diego, CA). Only follicles photographed in their entirety in one field were counted. Using the kidney sections, 20–25 glomeruli were imaged at a 20x magnification. The area of each glomeruli was measured and the number of nuclei where counted. Twenty glomeruli from each kidney were scored blindly as follows: 0, no glomerular lesions; 1, minimal thickening of mesangium; 2, noticeable increase in both mesangial and glomerular capillary cellularity; 3, presence of preceding conditions along with superimpose inflammatory exudate and capsular adhesion; 4, obliteration of the glomerular architecture included >70% of glomeruli. All the histological analysis was done in a blinded manner by a trained immune-toxicologist with extensive experience at characterizing histological evaluations.

### Immunofluorescent (IFC) and TUNEL Analysis

Paraffin sections (6 μm) were deparaffinized and rehydrated in an alcohol gradient. Sections were soaked in blocking buffer and washed with phosphate-buffered saline (PBS). After antigen retrieval, sections were incubated in the primary antibody, anti-Nitrotyrosine (N-tyr) (Novus Biologicals, clone: Em-30) at a 1:500 dilution at room temperature for an hour. For kidney sections, 20 glomeruli per mouse were used for quantification. Optimum photo settings were used to reduce background fluorescence, these settings were maintained for all photos. Using program Image J, the fluorescence-intensity density and area were measured for each glomerulus. For face sections, the whole length of the sample was photographed, including the epithelial layer and a portion of the dermis, at 20x magnification. Using Image J, N-tyr in four areas of the skin was quantified, areas of interest included; above the epithelial layer, the epithelium, the dermis, and hair follicles. Hair inside of the follicle was not measured due to non-specific binding. For kidney sections, images were taken at 20x magnification. For each mouse, 20 glomeruli were measured for fluorescence intensity using Image J and the area measured using the software on the Echo Revolve microscope. The fluorescence for all 20 glomeruli was averaged. Apoptotic cells in the facial tissue were visualized using the Click-iT Plus TUNEL Assay (Invitrogen). Paraffin sections (6 μm) were deparaffinized and rehydrated in an alcohol gradient. The Click-iT procedure was followed as per manufacturers specifications. The whole length of the skin sample was photographed, including the epithelial layer and a portion of the dermis, at 20x magnification. Positive TUNEL staining was considered when the fluorescent signal was more than 50% of the nuclei. The area and number of positive cells in three areas of the skin, the epithelium, dermis, and hair follicles. Positive staining present on the inner portion of the hair was considered non-specific binding.

### Plasma Protein Measures

At necropsy blood was collected from mice by cardiac puncture and placed into EDTA-tubes to prevent coagulation. Blood Samples were centrifuged and plasma was collected and frozen at −80°C until used. Cytokines and chemokines were measured using the V-PLEX Plus Pro-inflammatory Panel1 Mouse Kit from MSD (Rockville, MD). Plasma BAFF concentrations were measured using the Mouse BAFF/BLyS/TNFSF13B Quantikine ELISA Kit from R&D Systems (Minneapolis, MN) following the manufacturers protocol. Total IgG was quantified using IgG (Total) Mouse Uncoated ELISA kit (Invitrogen) following the manufacturers protocol. Anti-ds-DNA IgG present in the kidney was quantified using Mouse Anti-dsDNA IgG Antibody Assay Kit (Chondrex, inc.) following the manufacturers protocol. Plasma creatinine was measured using the Creatinine (serum) Colorimetric Assay Kit (Cayman Chemical; Ann Arbor, MI).

### Flow Cytometry Measures

Spleens were dissociated using the Spleen Dissociation Kit for mice (Miltenyi Biotec, Gladbach, Germany). Erythrocytes for both spleen and blood samples were removed using 1X Red Blood Cell Lysis Solution (Miltenyi Biotec). For cytokine secretion assays, cells were washed, counted, seeded at 4 × 10^5^cells/well in 200 ul. Cells were then given 1x eBioscience™ Cell Stimulation Cocktail (Invitrogen) and 1x eBioscience™ Protein Transport Inhibitor Cocktail (Invitrogen) incubated overnight, and stained for flow cytometry. Antibodies used for flow cytometry surface staining are as follows: anti-CD3 (145-2C11, ThermoFisher), anti-CD4 (GK1.5, ThermoFisher), and anti-CD8 (53-6.7, ThermoFisher). Cells were fixed with 4% formalin in PBS and permeabilized with 0.5% Tween20 in PBS. Antibodies used for intracellular staining are as follows: anti-IFN-γ (XMG1.2, ThermoFisher), anti-TNF-α (MP6-XT22, ThermoFisher), anti-IL-10 (JES5-16E3, ThermoFisher), and anti-IL-17 (eBio17B7, ThermoFisher). For T cell activation measures the following antibodies were used for white blood cells (WBCs) and splenocytes: anti-CD45 (REA737, Miltenyi); anti-CD3 (REA606, Miltenyi); anti-CD4 (REA604, Miltenyi); anti-CD8b (REA793, Miltenyi); anti-CD69 (REA937, Miltenyi). The right kidney was dissociated with the Multi Tissue Dissociation Kit 2 (Miltenyi Biotec). Cells were then stained with anti-CD45 (REA737, Miltenyi), anti-CD31 (REA784, Miltenyi), and intercellularly with anti-N-Tyr (1A6, Millipore). BM cells were stained for Mesenchymal stem cells (MSCs) using the following antibodies; anti-CD45 (REA737, Miltenyi), anti-CD105 (REA1058, Miltenyi), anti-CD29 (REA1074, Miltenyi), and anti-Sca-1 (REA422, Miltenyi). The gating strategy is outlined in Supplemental Figure 1.

### Stem Cell *in vitro* Culture

Femurs were excised from the animals at necropsy and cleaned of muscle and connective tissue. The femurs were then flushed with 2% FBS/2x PenStrep/DPBS to remove the bone marrow (BM). The cell solutions were washed, counted, and resuspended at 5^*^10^6^ cells/mL. MesenCult Basal Medium (Mouse) from StemCell Technologies (Cambridge, MA) was supplemented with a bottle of Mesencult™ 10x Supplement (Mouse) from Stemcell Technologies, 2 mL of the complete media were added to each well of a 24 well-plate along with 100 μL of diluted cells from each respective sample. The plates were incubated for 8 days at 37°C and 5% CO_2_. On day 8, the colony forming units (CFU) were counted by light microscopy by a blinded researcher. Hematologic progenitors were also measured in the BM samples resuspended at 5^*^10^6^ cells/mL cultured in MethoCult™ GF M3434 (Mouse) from StemCell Technologies (Cambridge, MA); 0.5 mL of the complete media was added to each well of a 24 well-plate along with 20 μL of diluted cells from each respective sample. The plates were incubated for 12 days at 37°C and 5% CO_2_. On day 7 and 12, the colonies were counted by light microscopy by a blinded researcher.

### Statistical Analysis

GraphPad Prism version 8.3.0 for Mac OS X (GraphPad Software, San Diego, CA, USA) was used to analyze the data. Two-tailed *t*-Test, and in some cases the ANOVA- Kruskal-Wallis test, were used to compare data. The level of statistical significance was set at 5%. Data are expressed as mean value ± standard error of the mean (SEM).

## Results

### Phenotypic Markers of SLE Pathology Were Ameliorated by RAS Modulation in MRL*-lpr* Mice

**Figure 1:**
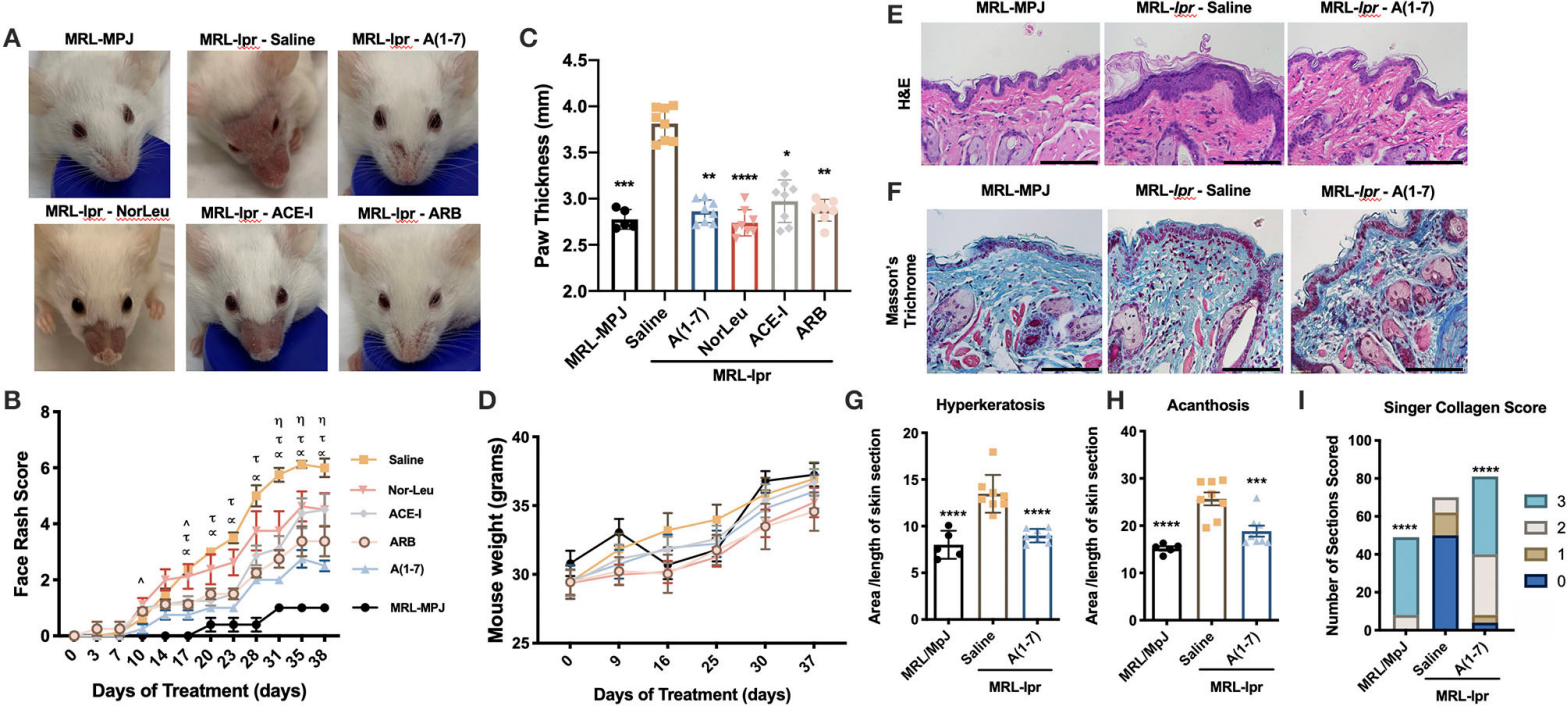
Pathologies associated with SLE are ameliorated with RAS modulation. MRL*-lpr* mice were treated with either saline, A(1-7), NorLeu, ACE-I or ARB; MRL-MpJ mice were used as healthy controls. **(A,B)** Skin lesions were scored every 3–5 days according to the following criteria: 0, not noticeable rash; 1, little redness, no hair loss, or inflammation; 2, minimal rash, little hair loss, or inflammation; 3, moderate rash, increased hair loss, light inflammation; 4, pronounced rash, near total hair loss, and obvious inflammation; 5, Rash is traveling up the face; 6, obvious wound above nose. Statistical significance is expressed by group A(1-7) (α), NorLeu (η), ACE-I (∧), and ARB (τ). **(C)** Paw thickness was measured at the end of the study using a caliper. **(D)** Mice were weighed weekly throughout the study. **(E,F)** Tissue taken from the snout was stained using H&E and Masson's Trichrome, bars represent 110 μm. **(G)** Hyperkeratosis was measured by dividing the area of the stratum corneum by the length of the tissue. **(H)** Acanthosis was measured by dividing the area of the stratum Basale/stratum spinosum by the length of the tissue. **(I)** The collagen was scored using the Singer Collagen Score as fallows: 3, Normal; 2, Abnormal collagen in the papillary dermis; 1, Abnormal collagen in the upper reticular dermis only; 1, Abnormal collagen in the upper and lower half of the reticular dermis. Statistical analysis was done using Prism 8.4.0, *t*-tests were used to compare all groups to saline treated MRL*-lpr* mice, except in the paw thickness where the ANOVA-Kruskal-Wallis test was used and the collagen score where the Chi-square test was used; *p ≤ 0.05, **p ≤ 0.01, ***p ≤ 0.001, ****p ≤ 0.0001.

During the study, the MRL/MpJ control mice had no signs of any skin lesions at any point during the study. In contrast, the MRL*-lpr* mice treated with saline developed an extensive rash that traveled from their cheeks to the area above the nose and in some cases up to the ears (Figure 1A). The difference in scores for the face rash were significantly different from for the MRL/MpJ vs. the saline treated MRL*-lpr* mice starting at 10 days after the start of treatment (Figure 1B). However, treatment with all of the RAS-modifying therapies significantly reduced the severity of the rash [A(1-7) starting at 17 days to the end of study; NorLeu starting at 31 days to the end of study; ACE-I at day 10 and 17 till the end of study; ARBs starting at 17 days to the end of study] resulting in only mild inflammation and hair loss compared to the saline treated MRL*-lpr* mice. At the end of the study, right paw thickness as a measure of joint inflammation/edema was assessed. MRL*-lpr* treated with saline mice had a significantly more inflammation when compared to MRL/MpJ mice or any of the mice treated with RAS modifiers, despite not having a significant change in body weight (Figures 1C,D).

### Histological Evaluation of Skin Sections Show a Reduction in Dermal Tissue Damage With A(1-7) Treatment

Skin samples collected at necropsy for MRL/MpJ and MRL*-lpr* treated with saline or A(1-7), where we saw the greatest therapeutic effect, were stained with H&E staining (Figure 1E). Hyperkeratosis, a thickening of the outer protein layer of the skin which manifests clinically as scaliness, was measured by dividing the area of stratum corneum by the length of the tissue (Figure 1G). Acanthosis, thickening of the epidermal layer, was also measured by dividing the area of the stratum basale/stratum spinosum by the length of the tissue section (Figure 1H). Decreased malar rash is important not only for the quality of life of SLE patients, but it may also be indicative of overall reduction in disease pathologies. Both hyperkeratosis and acanthosis was significantly increased in the snout tissues from MRL*-lpr* mice treated with saline compared to the control MRL/MpJ mice and was significantly reduced by treatment with A(1-7). The same skin sections were stained with Trichrome staining to look at the collagen matrix (Figure 1F). The skin sections from A(1-7) treated mice had a significantly healthier collagen score than MRL*-lpr* mice treated with saline, and reflected a collagen matrix comparable to those of MRL/MpJ mice (Figure 1I). N-Tyr and TUNEL staining were also measured to evaluate cell death that may be caused by OS (Supplemental Figures 2, 3). It is important to note here that the MRL/MpJ do develop SLE-like pathologies but at a delayed timeline (33, 34). The oxidative stress in these tissues may be early signs of disease and may be comparable to that seen in MRL-*lpr* mice at this age or higher because of the damage that has already had a devastating effect on the skin. The hair follicles were the most affected by both OS and cell death in MRL*-lpr*-saline treated mice; this was reversed by A(1-7) treatment. TUNEL staining in the dermis, follicles, and overall correlated with the face scores at necropsy. Since follicles are the most affected, and they hold a stem cell niche in the skin, this may point to the importance of this population in preventing skin inflammation.

### A(1-7) Modulates Immune Parameters More Than Other RAS Modifiers

**Figure 2:**
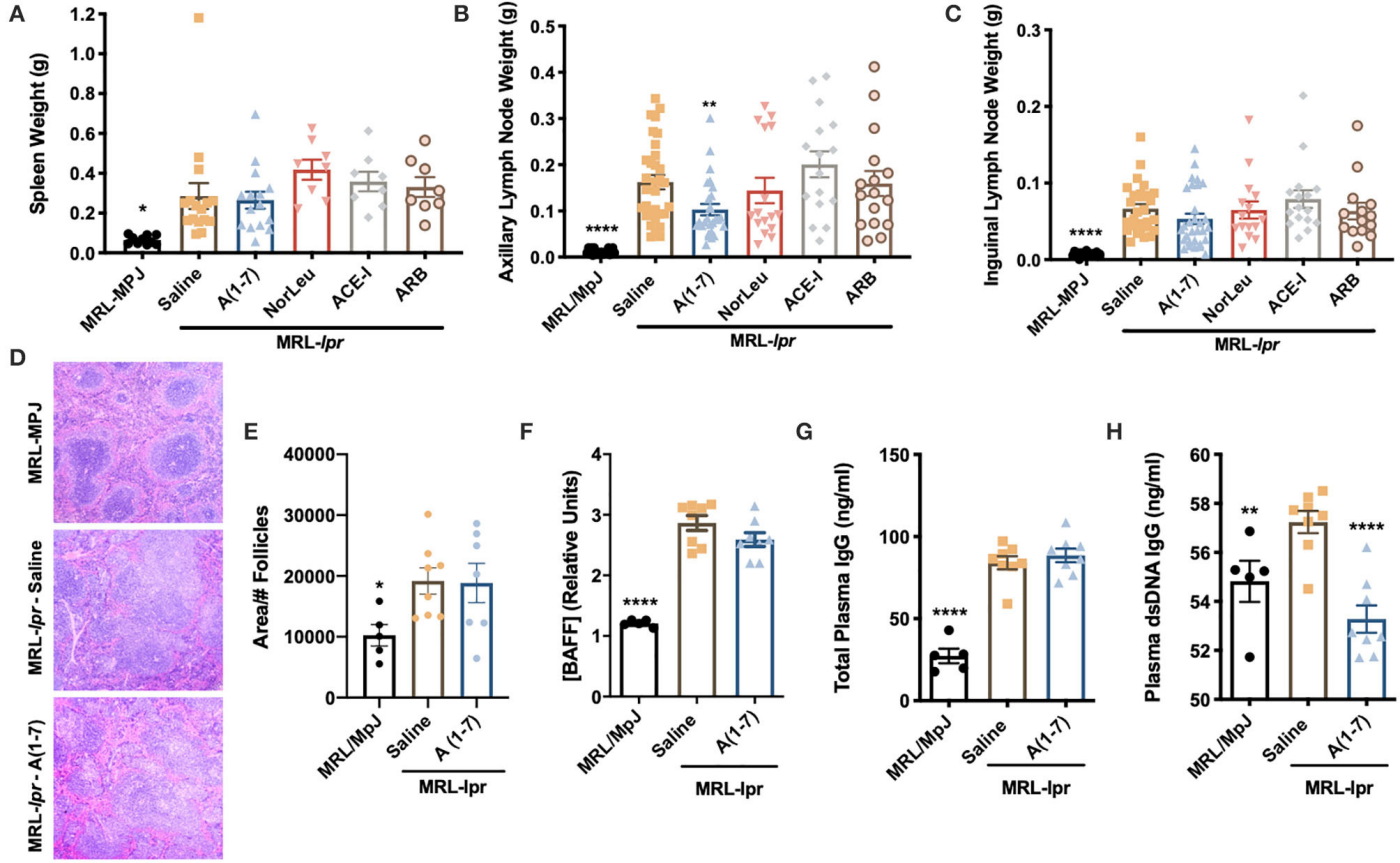
A(1-7) treatment shows some improvement in reducing lymph node size and anti-dsDNA IgG antibodies. **(A–C)** The spleen and the auxiliary and inguinal lymph nodes were removed at necropsy and weighed. **(D)** Paraffin-embedded spleen samples were stained with H&E. The whole span of the spleen was photographed at x20 magnification. **(E)** Only follicles completely visible in the field where counted and measured. **(F–H)** The concentration of circulating BAFF, total plasma IgG, and anti-dsDNA IgG antibodies were measured from plasma collected at necropsy. Statistical analysis was done using Prism 8.4.0, *t*-tests were used to compare all groups to saline treated MRL*-lpr* mice; *p ≤ 0.05, **p ≤ 0.01, ****p ≤ 0.0001.

Secondary lymphoid organs (spleens, axillary, and inguinal lymph nodes) were collected and weighed at necropsy. MRL*-lpr* mice had a significantly larger spleen compared to MRL-MpJ mice, none of the RAS treatments reduced this (Figure 2A). Axillary and inguinal lymph nodes were larger in MRL*-lpr* mice compared to MRL/MpJ control animals (Figures 2B,C). A(1-7) was the only treatment that significantly reduced axillary lymph node size and had the most effect in reducing inguinal lymph node size, although this was not significant. Spleen sections were stained by H&E and analyzed for average follicle size (Figure 2D). MRL*-lpr* mice had larger splenic follicle size and A(1-7) treatment had no significant effect (Figure 2E). Circulating cytokines were measured from the plasma of MRL/MpJ and MRL*-lpr* mice treated with saline or A(1-7) (Supplemental Figure 4). There were significant changes in both pro- and anti- inflammatory cytokines between the control groups, but there were no significant changes observed with A(1-7) treatment. Plasma BAFF levels were also measured (Figure 2F). Again, we saw a significant increase of BAFF plasma levels in MRL*-lpr* mice and A(1-7) treatment had no effect. Total IgG and anti-dsDNA-IgG antibodies in the plasma were also higher in MRL*-lpr* mice and A(1-7) only had an effect in lowering anti-dsDNA-IgG antibodies (Figures 2G,H). Again, MRL/MpJ here also have a considerable amount of anti-dsDNA-IgG antibodies but less than MRL*-lpr* mice because they are still early in disease onset.

### T Cell Activation Is Reduced by RAS Modulation in MRL*-lpr* Mice

**Figure 3:**
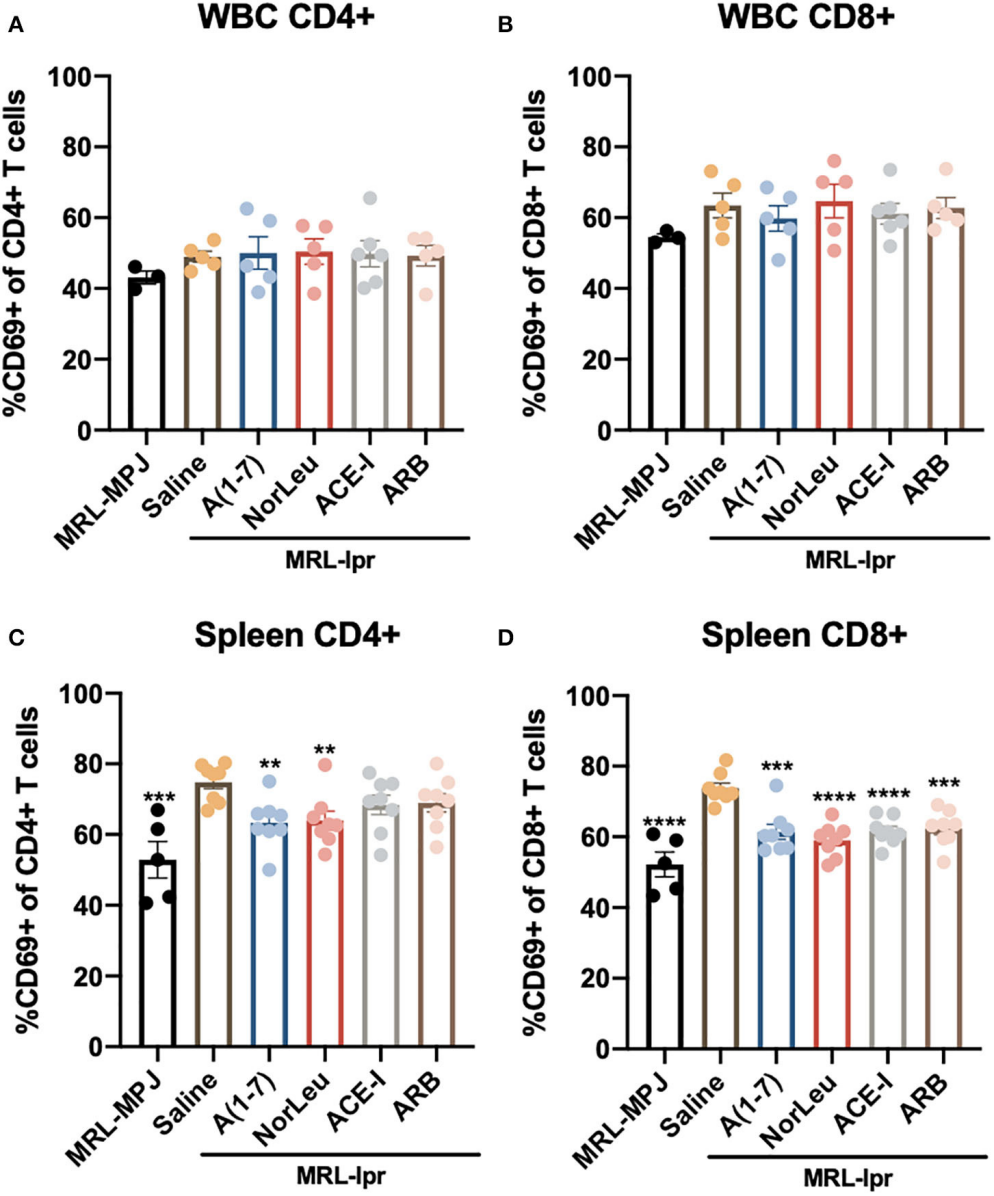
Splenic but not circulating T cell activation is affected in MRL*-lpr* mice, and rescued with RAS-modulating therapies. At necropsy, splenic cells, and WBCs were isolated and stained for T cell markers (CD3, CD4, CD8) and the T cell activation marker CD69. Samples were then analyzed by flow cytometry. T cells were identified as CD45^+^ CD3^+^ and then either CD4^+^ or CD8^+^. The gating strategy for the T cell characterization can be found in Supplemental Figure 5. CD69 was measured in both CD4 and CD8 T cells in the blood **(A,B)** and in the spleen **(C,D)**. Statistical analysis were done using Prism 8.4.0, *t*-tests were used to compare all groups to saline treated MRL*-lpr* mice; **p ≤ 0.01, ***p ≤ 0.001, ****p ≤ 0.0001.

MRL*-lpr* mice have an unusual phenotype where they contain a significant amount of double negative CD3^+^CD4^−^CD8^−^ T cells and because of this MRL*-lpr* mice have significantly lower percentage of CD4^+^ T cells, CD8^+^ T cells in both the spleen and circulation (Supplemental Figure 5). The CD4/CD8 ratio is not affected in the blood of MRL*-lpr* mice but it is significantly higher in the spleen. T cell CD4 and CD8 levels or ratios were not affected with any of the RAS treatments. However, RAS modulation has a significant effect T cell activation was measured by expression of CD69 in both peripheral WBCs and splenocytes (Gating strategy in Supplemental Figure 6) (Figure 3). A significant increase of activated T cells was measured in the spleen of saline treated MRL*-lpr* mice but not in circulating T cells. A(1-7) and NorLeu reduced the level of both activated CD4^+^ and CD8^+^ T cells in the spleen. ACE-I and ARBS were only able to reduce the level of CD8^+^CD69^+^ T cells and not CD4^+^CD69^+^ T cells.

### Treatment With RAS Modulators Significantly Affected Both Pro- and Anti-inflammatory Cytokine Production in Several Populations of Stimulated T-Cells From the Spleen

**Figure 4:**
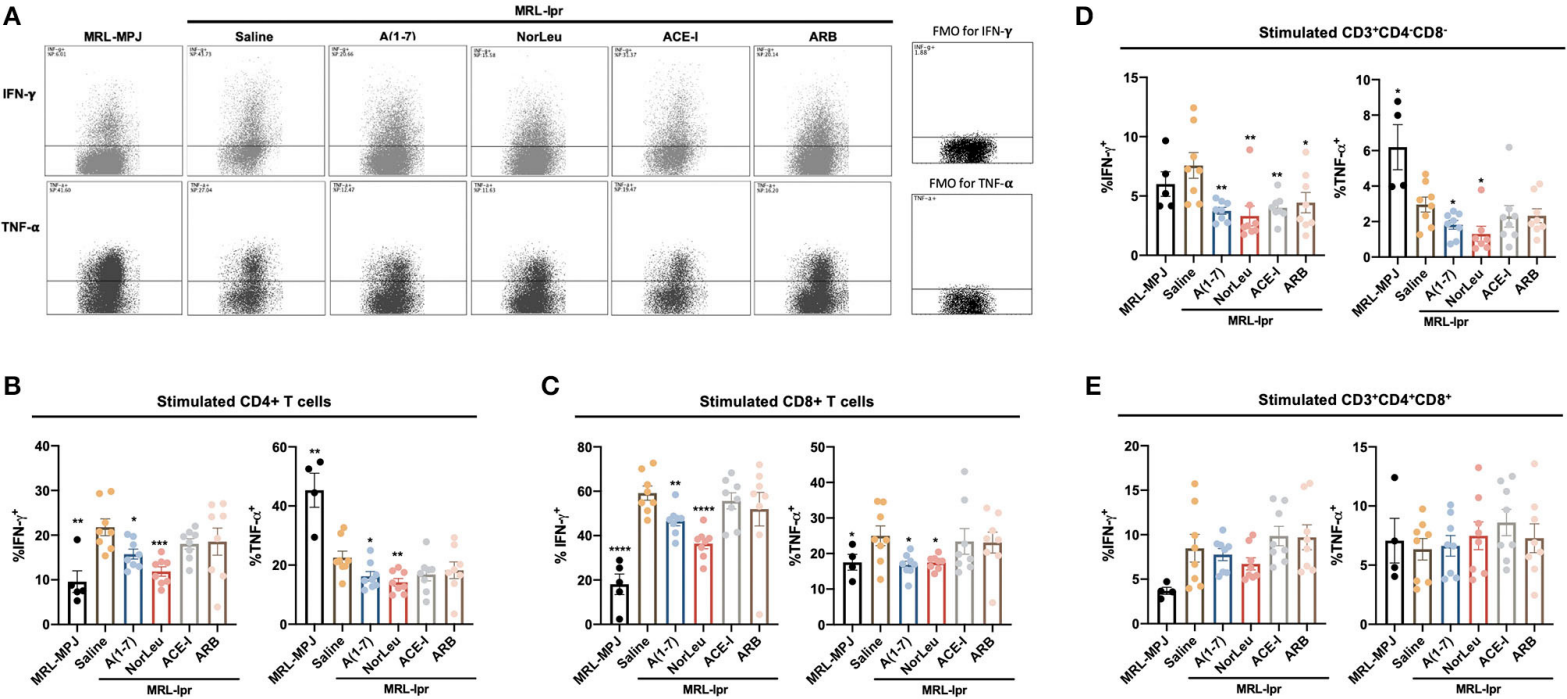
T cells producing pro-inflammatory cytokines are seen in higher levels in MRL*-lpr* mice and reduce with A(1-7) and NorLeu treatment. Splenic cells were stimulated for 16 h with PMA/ionomycin and then stained with T cell markers and for IFN-γ and TNF-α production. The gating strategy for the T cell characterization can be found in Supplemental Figure 7. **(A)** Representative plots show samples of CD4^+^ Tcells expressing IFN-γ and TNF-α after stimulation. The percent of **(B)** CD4^+^ and **(C)** CD8^+^ T cells that express IFN-γ and TNF-α were measured. The percentage of cells producing IFN-γ and TNF-α in the unusual **(D)** CD3^+^CD4^−^CD8^−^ and **(E)** CD3^+^CD4^+^CD8^+^ populations were also measured. Statistics were run using Prism 8.4.0, *t*-tests were used to compare all groups to saline treated MRL*-lpr* mice; *p ≤ 0.05, **p ≤ 0.01, ***p ≤ 0.001, ****p ≤ 0.0001.

**Figure 5:**
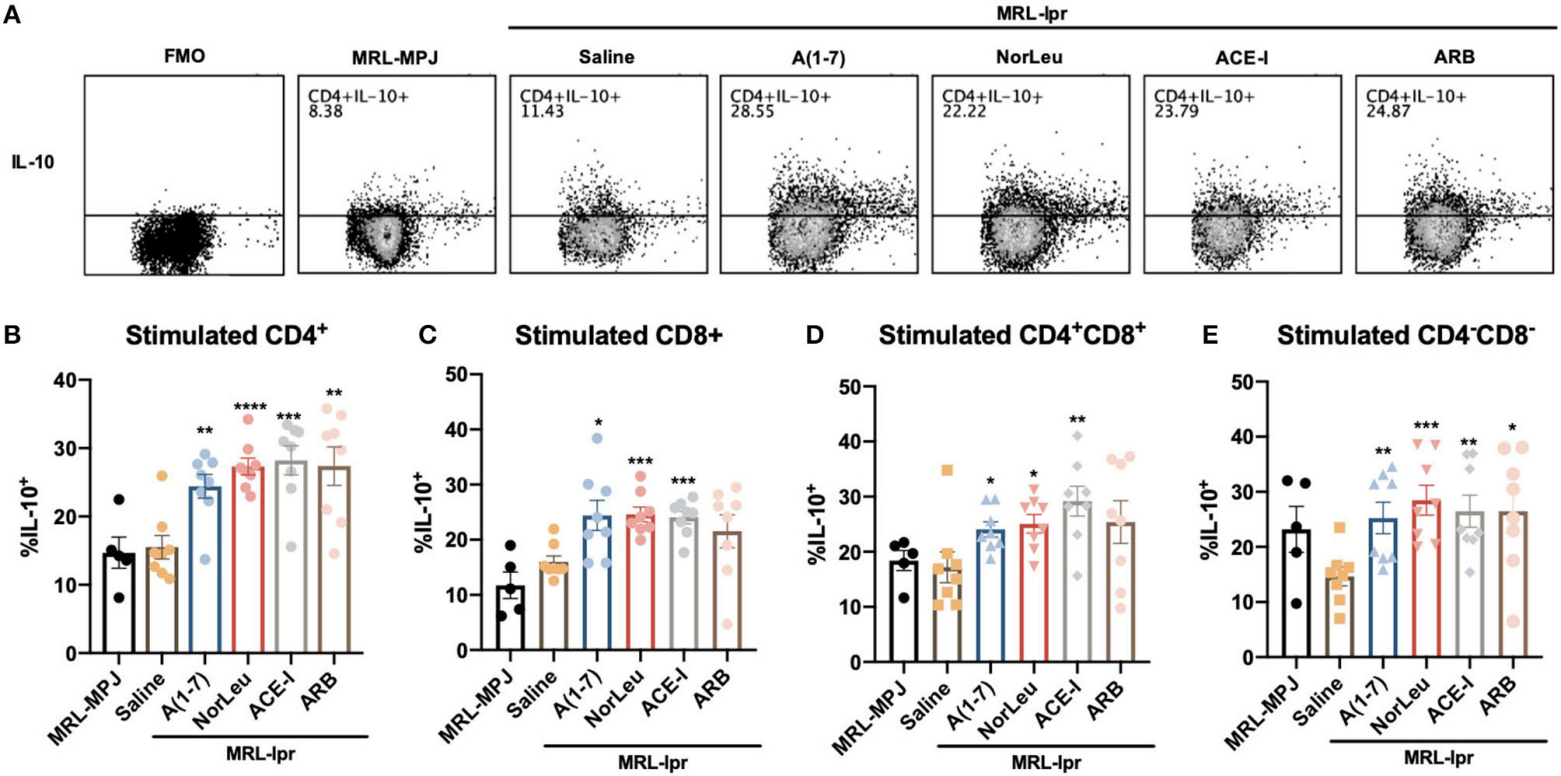
IL-10 producing T cells are increased with RAS-modifying treatments. Splenic cells were stimulated for 16 h with PMA/ionomycin and then stained with T cell markers and for IL-10 production. The gating strategy for the T cell characterization can be found in Supplemental Figure 7. **(A)** Representative plots show samples of CD4^+^ T cells expressing IL-10 after stimulation, and an example of fluorescent minus one (FMO). The percent of **(B)** CD4^+^ and **(C)** CD8^+^ T cells that express IL-10 were measured. The percentage of cells producing IL-10 in the unusual **(D)** CD3^+^CD4^−^CD8^−^ and **(E)** CD3^+^CD4^+^CD8^+^ populations were also measured. Statistics were run using Prism 8.4.0, *t*-tests were used to compare all groups to saline treated MRL*-lpr* mice; *p ≤ 0.05, **p ≤ 0.01, ***p ≤ 0.001, ****p ≤ 0.0001.

Upon stimulation with PMA/ionomycin, both CD4+ T-cells and CD8+ T cells from MRL*-lpr* mice treated with A(1-7) and NorLeu showed decreased production of IFN-γ and TNF-α (Figure 4A, Supplemental Figure 7-gating strategy), compared to saline treatment. Interestingly, the sample with the highest amount of TNF-α producing CD4+ T cells was the MRL/MpJ group, which may be again explained by the delayed but present pathology in this mouse model. No change was seen in ACE-I and ARB treatment groups (Figures 4B,C). IFN-γ production in stimulated double negative CD3^+^CD4^−^CD8^−^ T cells from MRL*-lpr* mice spleen was significantly decreased in all RAS treatment groups compared to saline treatment. TNF-α production in the same population of T cells was significantly decreased with treatment with A(1-7) and NorLeu, but not ACE-I and ARB (Figure 4D), compared to treatment with saline. Stimulated double positive CD3^+^CD4^+^CD8^+^ T cells showed no change in TNF-α or IFN-γ levels among MRL-MPJ mice, saline-treated MRL*-lpr* mice, and all RAS treatment groups (Figure 4E). In stimulated spleen CD4^+^ T cells and CD8^+^ T cells, compared to saline-treated MRL*-lpr* mice, IL-10 production was significantly increased in A(1-7), NorLeu, and ACE-I treatment groups. ARB significantly increased IL-10 in CD4^+^ T cells but not in CD8^+^ T cells (Figures 5A–C). Stimulated T cells that were double-positive CD3^+^CD4^+^CD8^+^ treated with A(1-7), NorLeu, and ACE-I all significantly increased production of IL-10 compared to saline-treated MRL*-lpr* mice; treatment with ARB showed no change (Figure 5D). Double-negative CD3^+^CD4^−^CD8^−^ stimulated T cells treated with RAS modulators showed significant increase in IL-10 production in all RAS treatment groups, compared to saline-treated MRL*-lpr* mice (Figure 5E). Overall, treatment with RAS modulating therapies support a reduction in inflammatory T cell profiles.

### OS and Inflammation in the Kidney Were Reduced by Treatment With RAS Modifying Treatment

**Figure 6:**
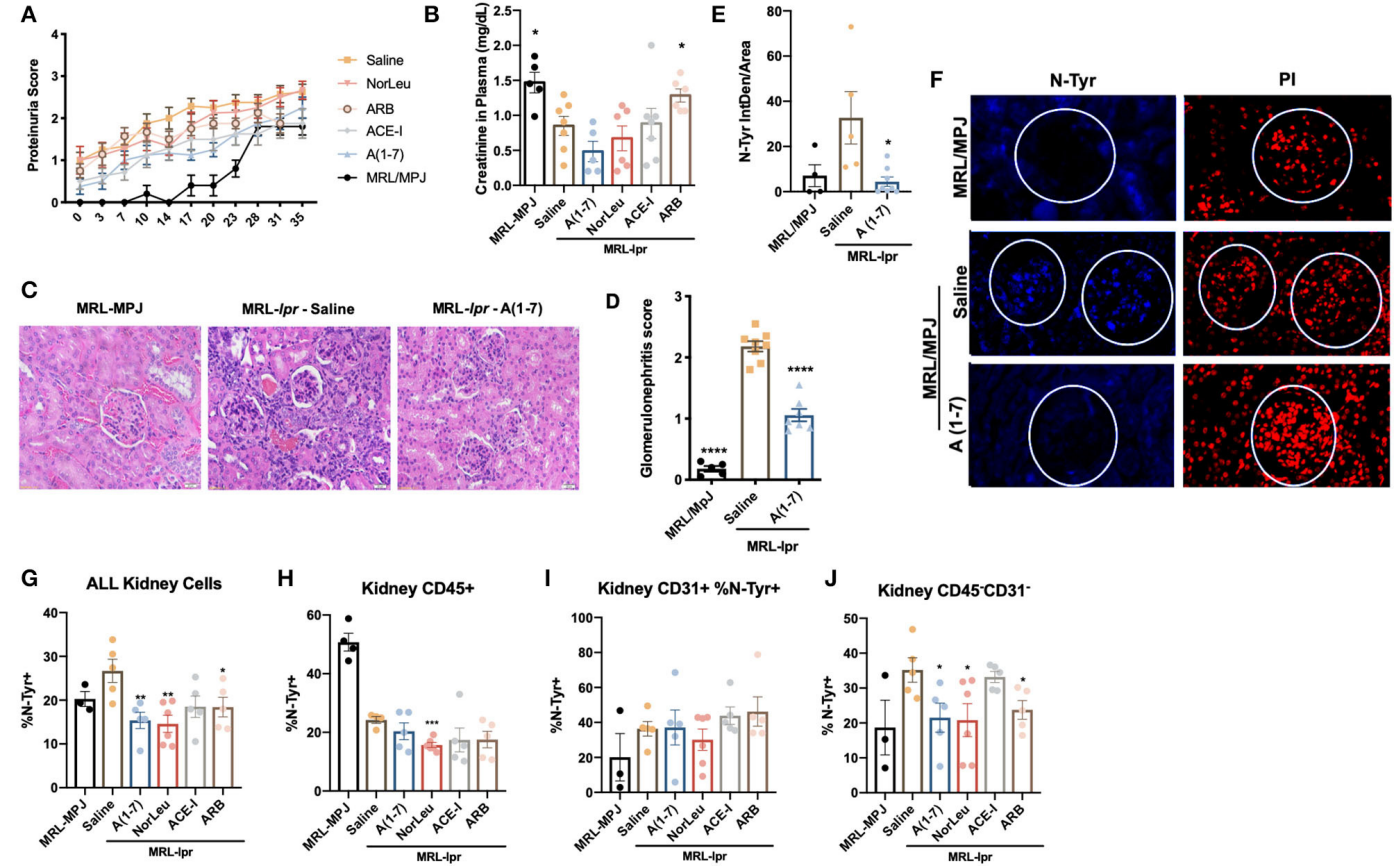
RAS-modulation, especially A(1-7) treatment, improves kidney health. **(A)** Protein scores were taken every 3–4 days throughout the study duration, scores were: 0, none; 1, trace; 2, >30 mg/dl; 3, >100 mg/dl. **(B)** At necropsy, plasma was collected and creatinine levels were measured. **(B)** Kidney samples from both MRL-Mpj and MRL-*Ipr* mice where stained using H&E, photos were taken at x20 magnification. **(C)** Twenty glomeruli from each kidney were scored as follows: 0, no glomerular lesions; 1, minimal thickening of mesangium; 2, noticeable increase in both mesangial and glomerular capillary cellularity; 3, presence of preceding conditions along with superimpose inflammatory exudate and capsular adhesion; 4, obliteration of the glomerular architecture included >70% of glomeruli. **(D)** Paraffin-embedded kidney sections were stained with anti-N-Tyr-Ab(blue) and counterstained with PI (red) antibodies to measure oxidative stress. **(E)** Photos were taken at x20 magnification using Echo Revolve microscope and the intensity inside the glomerulus was measured using Image **(F)**, and corrected to total area of the glomerulus; each data point represents one animal and the average from 20 glomeruli per animal. At necropsy kidneys we dissociated and stained with anti-N-Tyr-Ab (All, **G**) intercellularly and extracellularly for immune cells (CD45^+^, **H**), endothelial (CD31^+^, **I**) and kidney (CD45^−^CD31^−^, **J**). Statistics were run using Prism 8.4.0, *t*-tests were used to compare all groups to saline treated MRL*-lpr* mice; *p ≤ 0.05, **p ≤ 0.01, ***p ≤ 0.001, ****p ≤ 0.0001.

Although there was a significant difference in proteinuria scores in the MRL/MpJ compared to the saline treated MRL-*lpr* mice, no significant change was seen with treatment (Figure 6A). Plasma creatinine levels, measured at the end of study, show a significant increase in only the MRL/MpJ mice, and the MRL*-lpr* ARB treated mice compared to the MRL*-lpr*-saline treated mice (Figure 6B). A(1-7) did have the lowest levels of plasma creatinine, but the changes were not significant. Histology showed a significant increase glomerular nephritis in the MRL*-lpr* mice treated with saline and A(1-7) decreased this histological measure of kidney pathology (Figures 6C,D). IFC for N-tyr staining of the kidney shows a significant increase in oxidative damage in the glomerulus in the MRL*-lpr* mice that was reduced in mice treated with A(1-7) (Figures 6E,F). At necropsy, kidneys were also digested into a single cell suspension and stained for markers that denote immune cells (CD45^+^, Figure 6H), endothelial cells (CD31^+^, Figure 6I) and other cells of the kidney (CD45^−^CD31^−^, Figure 6J). N-tyr was again used to stain these cells for oxidative damage (Gating strategy in Supplemental Figure 8). N-tyr staining in the kidney overall shows a significant decrease in OS with A(1-7), NorLeu, or ARB treatment (Figure 6G). This change is driven by reduced N-tyr staining in the kidney cells (glomerular, tubal, etc.) rather than the immune or endothelial cells found in the kidney.

### RAS Modification Rescues BM Health in MRL*-lpr* Mice

**Figure 7:**
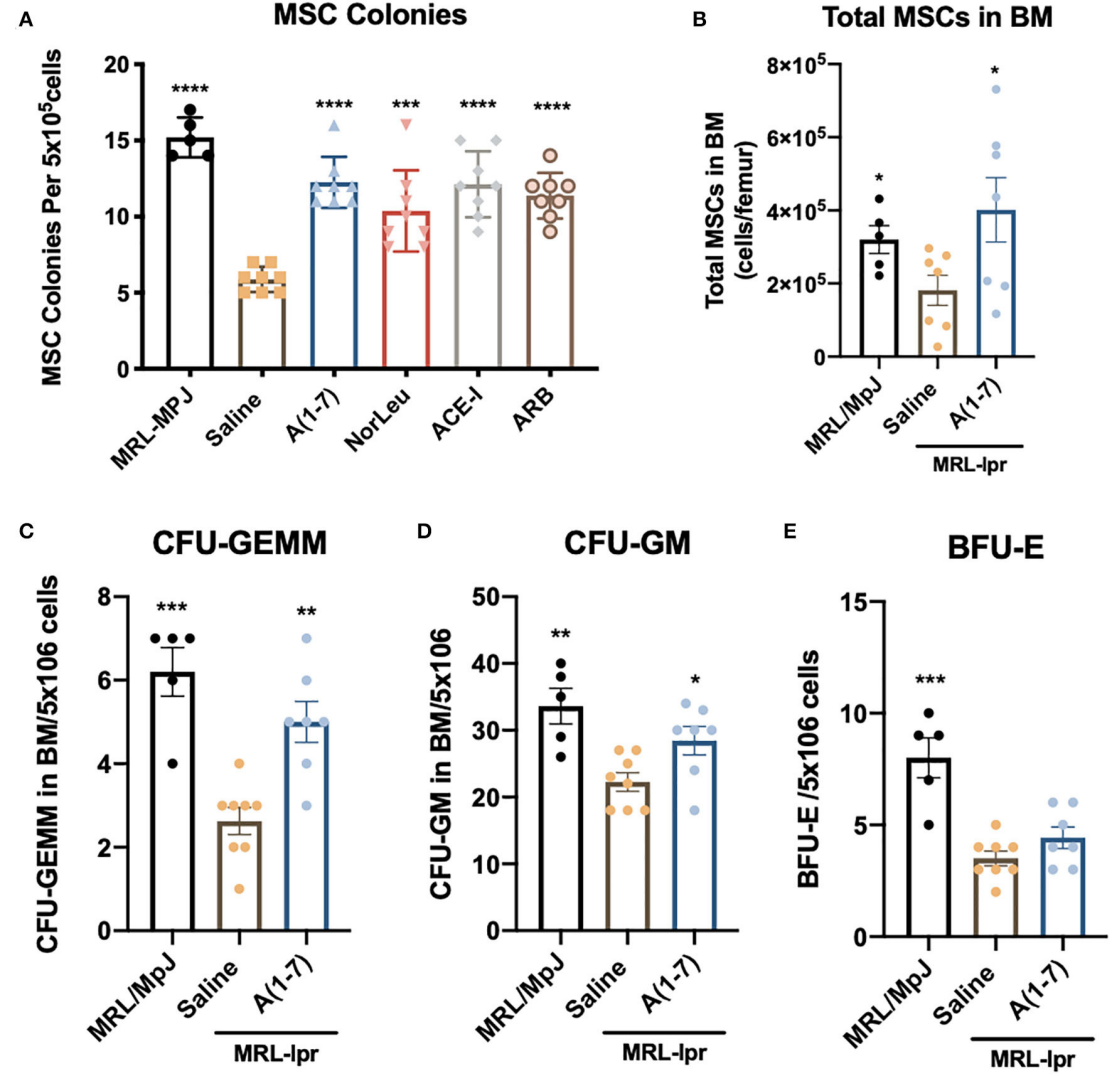
BM stem cells are diminished in MRL*-lpr* mice and rescued by RAS modulation. BM from the both femurs was collected at necropsy. **(A)** Cells were plated on MesenCult™ media and 7 days later the number of MSC-CFUs was counted. **(B)** Flow cytometry was used to measure the number of MSCs (CD45^−^CD29^+^Sca-1^+^CD105^+^) per femur, the gating strategy is outlined in Supplemental Figure 1. BM cells were also plated on MethoCult™ media and the CFU-GEMM **(C)** CFU-GM **(D)** and BFU-E **(E)** were measured as per manufacturer's instructions. Statistics were run using Prism 8.4.0, *t*-tests were used to compare all groups to saline treated MRL*-lpr* mice; *p ≤ 0.05, **p ≤ 0.01, ***p ≤ 0.001, ****p ≤ 0.0001.

BM samples from MRL*-lpr* mice treated with saline had a significant reduction in MSCs as measured by colony forming units (CFU) in MesenCult media (Figure 7A). RAS modification by either A(1-7), NorLeu, ACE-I, or ARB treatment resulted in nearly normal levels of MSC-CFUs. MSCs were also measured by flow cytometry (CD45^−^, CD29^+^, Sca-1^+^, CD105^+^) (Figure 7B). Again, there was a significant decrease in MSCs measure in MRL*-lpr* mice which were rescued by A(1-7) treatment. Hematopoietic progenitors in the BM were also measured in using MethoCult media. CFU-GEMM and CFU-GM are progenitor cells that ultimately give rise to monocytes and neutrophils. CFUs for both of these cell populations were decreased in MRL*-lpr* mice treated with saline, but not in the BM of those treated with A(1-7) (Figures 7C,D). BFU-E colonies, which ultimately give rise to erythrocytes, were also depleted in MRL*-lpr* mice but were not rescued by A(1-7) treatment (Figure 7E).

## Discussion

Although current therapies have extended the life of most SLE patients, there is still a significant discrepancy in life expectancy (35). The leading causes of death in these patients has shifted from early complications of SLE to cardiovascular disease, infections, and renal disease that occur later in life. Therapeutics used in these patients may account for some of these complications that arise in late life, but there is likely also a contribution from the underling disease. OS and inflammation are well-characterized in SLE and are also major contributors to cardiovascular disease and renal disease (36, 37). Results here show that A(1-7), NorLeu and ARBs, not ACE-Is, are able to reduce OS stress in the kidney, perhaps leading to reduced tissue injury. Both A(1-7) and NorLeu treated animals consistently display lower levels of T cell activation and reactivity. Data from our lab has shown that A(1-7) reduces OS and inflammation caused by diabetes in mouse bone marrow (31). In LPS-induced models of inflammation, A(1-7) treatment reduced the expression of pro-inflammatory cytokines (38). Our lab and others have demonstrate the cardio-protective and reno-protective effects of A(1-7) in a number of animal models (32, 39–46). Of note, several published and unpublished studies in our lab have shown different outcomes with A(1-7) vs. NorLeu treated mice in various disease models (47). The different results seen here with A(1-7) vs. NorLeu treated mice and those seen in past studies are likely due to differential binding patterns to all the known A(1-7) targets which include; AT1R (48, 49), AT2R (50–53), ACE (54–56), Mas (57), and MrgD (58).

A significant part of the SLE population consists of women of childbearing age (59), ACE-Is, and ARBs are contraindicated in this population as their use increases the likelihood of miscarriage and kidney problems in the children (60). Further, patients with early-onset SLE and without hypertension run the risk of progressing to hypotension. Although ACE-Is offer clinical evidence of the promises of modulating RAS for the treatment of SLE, their side-effects make them inappropriate or unsafe for chronic use in large portions of SLE patients. Both ACE I and ARBs increase circulating levels of A(1-7). Interestingly, a portion of the activities of ACE-I have been tied to their increase in circulating levels of A(1-7) potentially providing a therapeutic alternative to ACE-Is (61). ARBs may also engage the protective arm of RAS through increasing Ang-II binding to AT2 (62). Here we found that ACE-Is and ARBs have definite benefit that extends beyond the reduction of kidney damage, this is an important finding in that these medications are already available for prescription. However, the goal of this study was to highlight the potential of targeting the protective arm of RAS in order to develop therapies that do not have the same teratogenic and hypotensive inducing risks that are seen with ACE-Is and ARBs. Importantly, in several of the parameters tested A(1-7) and NorLeu had benefits beyond those provided by ACE-Is and ARBs.

The improvements seen with A(1-7) treatment may be partially explained by the improved MSC profile seen in these studies. MSCs have recently gained notoriety as a possible therapeutic option for SLE patients by reducing inflammation and OS (63, 64). Genetically induced MSC dysfunction has been correlated with SLE disease (65). Further this therapeutic model of allogenic transplantation of MSCs reduces pathologies associated with SLE (66). Our studies also show a decrease in number and clonability of these cells in MRL*-lpr* mice. Treatment with all of the RAS-modifying therapies improved the clonability of these cells. A(1-7) specifically, shows an increase in both number and clonality of these cell and other hematopoietic progenitors. Although we think that A(1-7) is acting by various mechanisms to improve SLE-related pathologies in these MRL-lpr mice, MSC health is likely one of them. This result may be a potential alternative to MSC transplantation if therapeutic intervention targeting RAS can be enough to improve BM health.

Results from our study show definite benefit in several SLE pathologies that are likely due to reducing oxidative damage, immune activation and BM dysfunction. Both A(1-7) and NorLeu have shown safety and efficacy in other diseases (67–69). Our results show that there is likely a protection of the tissue in this SLE model which in turn likely leads to lower antigen burden available to activate immune cells. Therefore, targeting the protective arm of the RAS provides a novel therapeutic paradigm in SLE that does not rely on immunosuppression.

## Data Availability Statement

All datasets generated for this study are included in the article/Supplementary Material.

## Ethics Statement

All animal studies have been reviewed and approved by both the University of Arizona and University of Southern California Institutional Animal Care and Use Committees (IACUC).

## Author Contributions

All of the authors contributed significantly to the work presented here. MS and KR contributed to the experiment planning and interpretation of data. MS, ND, and CH contributed to the execution of experiments. MS, ND, and CH wrote the manuscript. KR and MS edited the manuscript.

## Conflict of Interest

The authors declare that the research was conducted in the absence of any commercial or financial relationships that could be construed as a potential conflict of interest.
