# Supplementary material for: Targeting the Protective Arm of the Renin-Angiotensin System to Reduce Systemic Lupus Erythematosus Related Pathologies in MRL-lpr Mice
Source: Front Immunol. 2020 Jul 23;11:1572. doi: 10.3389/fimmu.2020.01572 (PMC7390909; doi:10.3389/fimmu.2020.01572)
Supplement: Supplementary file 1 [file Data_Sheet_1.docx]

Supplementary Material

#
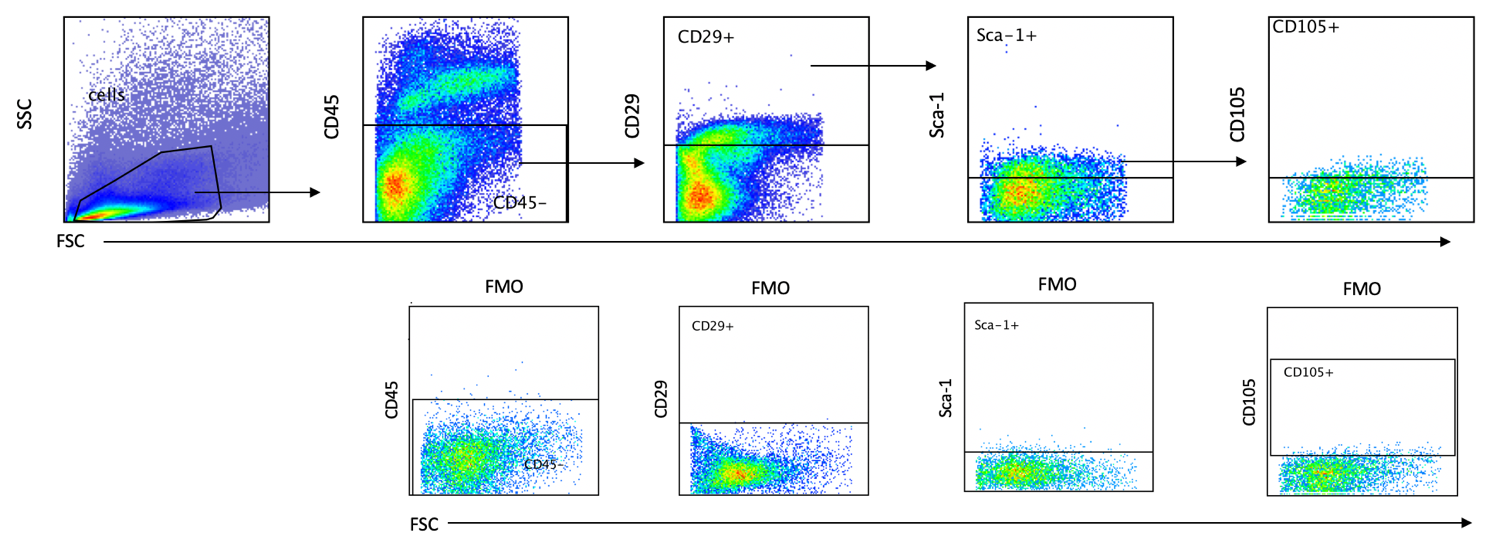
Supplementary Figure

**Supplemental Figure 1. Gating strategy for the analysis of MSCs in BM.** At necropsy BM cells were isolated and then stained with MSC cell markers (CD29, Sca-1 and CD105) and immune cell marker (CD45). MSCs were characterized as CD45-CD29+Sca-1+CD105+.


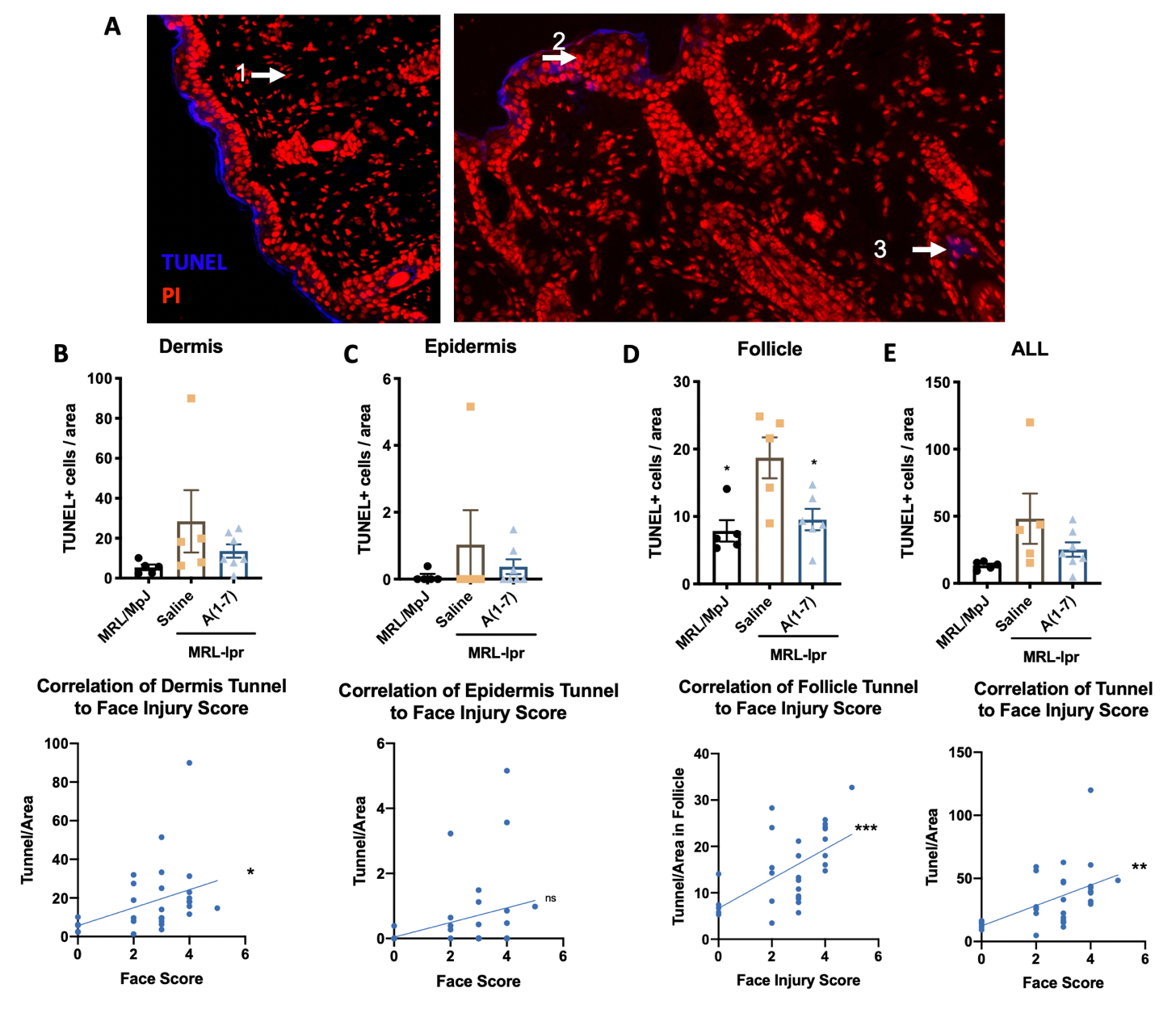


**Supplemental Figure 2. A reduction in apoptotic cells in the skin was measured, and correlated with facial scores at necropsy.** (**A**) Skin sections were stained using a TUNEL Assay (blue) and counter stained with PI (red). The whole span of the sample was photographed at x20 magnification; dermis (arrow 1), epidermis (arrow 2) and hair follicle (arrow 3) are marked by arrows. Cells that stained positive for N-tyr staining were counted and corrected for area of the section. Three areas of the skin where focused on the dermis (**B**), epidermis (**C**), hair follicle (**D**) and all the counts pooled together (**E**). The number apoptotic cells and were corrected against the area. Correlation between apoptotic cells and final facial scores measured in the dermis (**F**), epidermis (**G**), hair follicle (**H**) and all the counts pooled together (**I**). Statistics were run using Prism 8.4.0, t-tests were used to compare all groups to saline treated MRL*-lpr* mice, or in total samples for the correlation graphs; *p≤ 0.05, **p ≤ 0.01, ***p ≤ 0.001.

**
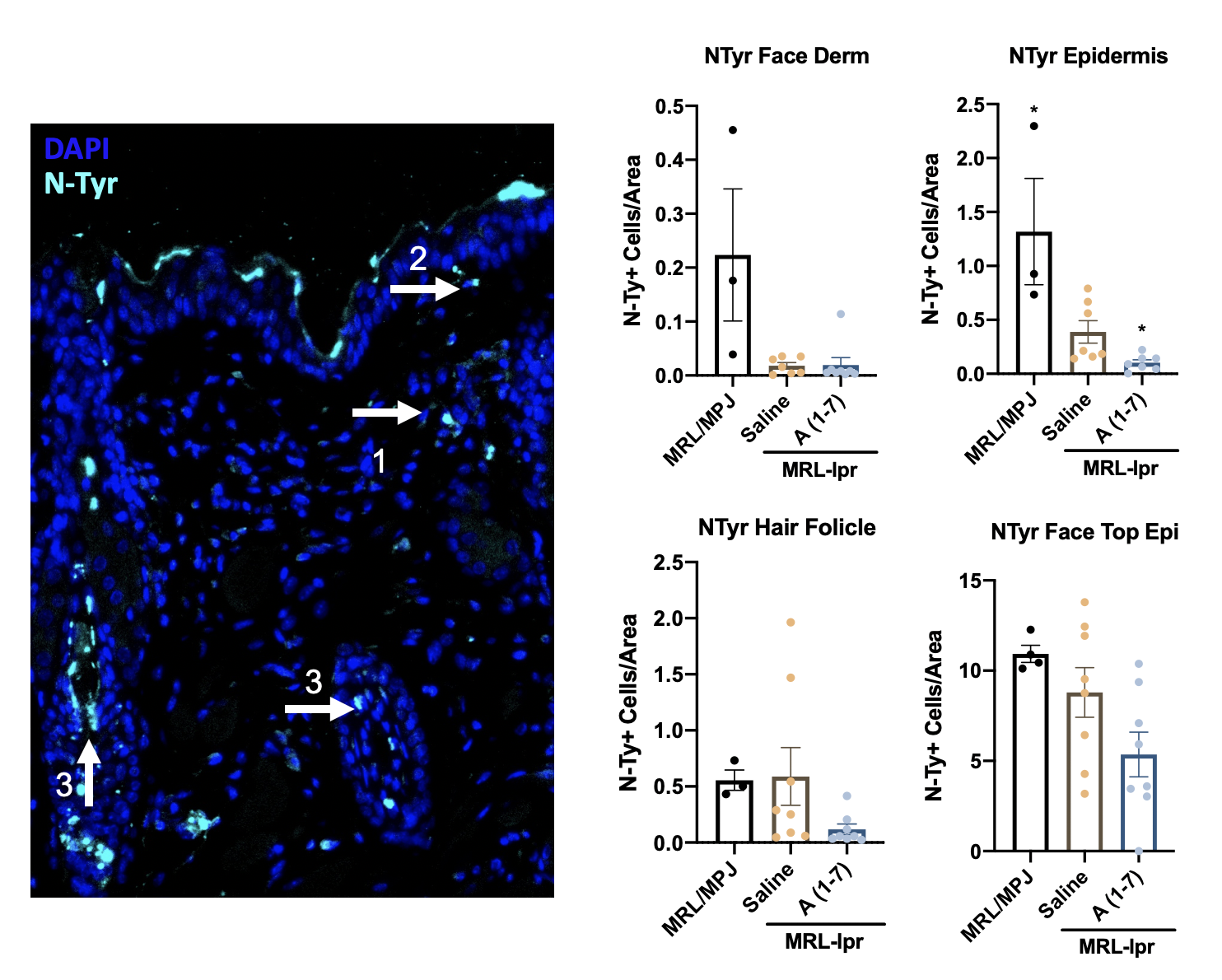
**

**Supplemental Figure 3. Increased levels of oxidative stress where seen in the MRL-MpJ mice in all areas of the skin and reduced with A(1-7) treatment.**  Skin sections were stained with anti-N-tyr (aqua) antibodies and counter stained with DAPI (blue). (**A**) The whole span of the sample was photographed at x40 magnification. Cells that stained positive for N-tyr staining were counted and corrected for area of the section. Three areas of the skin focused on the dermis-1 (**B**), epidermis-2 (**C**), hair follicle-3 (**D**) and all the counts pooled together (**E**). Statistics were run using Prism 8.4.0, t-tests were used to compare all groups to saline treated MRL*-lpr* mice; *p≤ 0.05.

**
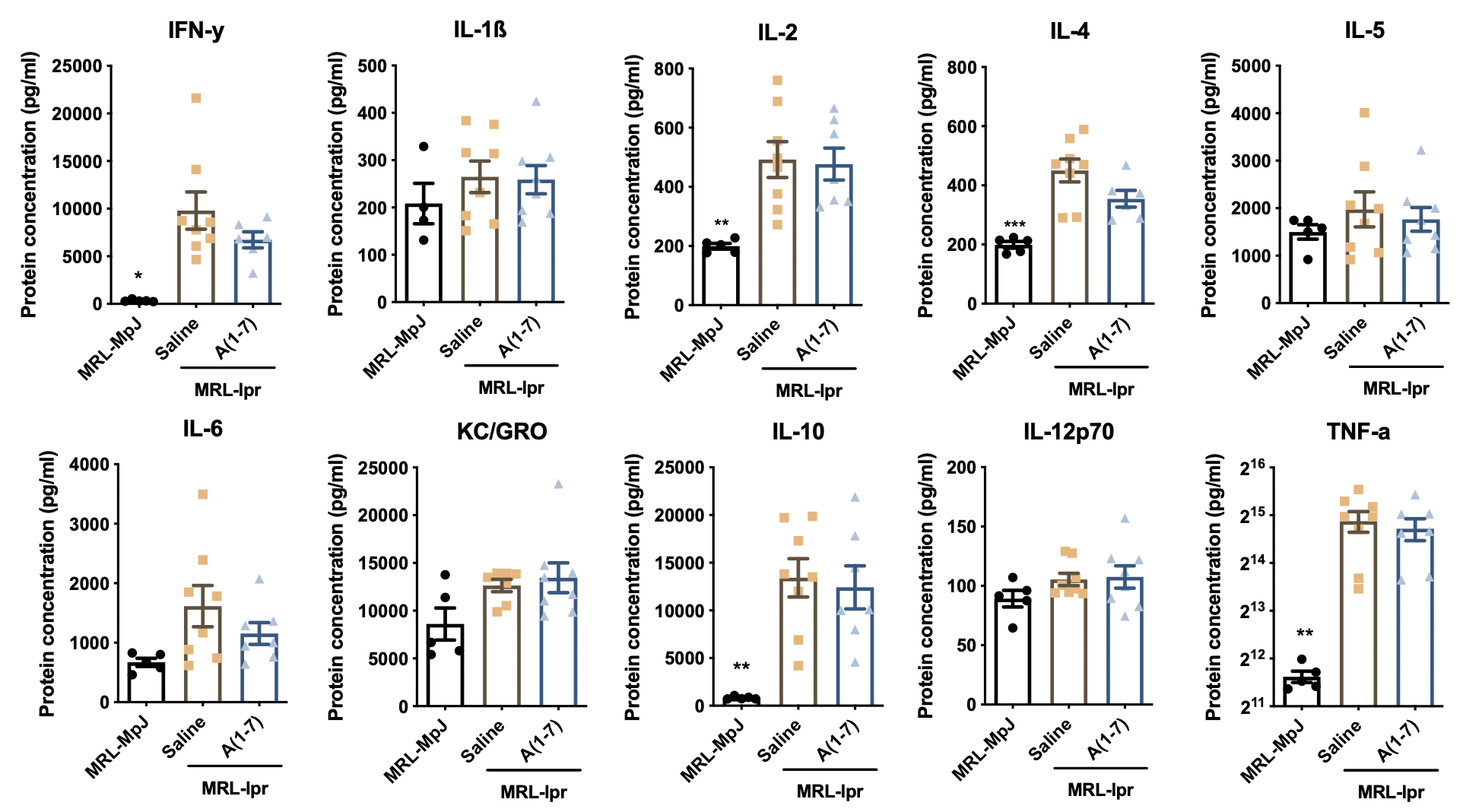
 Supplemental Figure 4. Circulating cytokines/chemokines are significantly different in MRL-MpJ vs MRL*-lpr* mice, but are not affected by treatment with A(1-7).** Plasma was collected at necropsy. Cytokines and chemokines were measured using the V-PLEX Plus Pro-inflammatory Panel1 Mouse Kit from MSD (Rockville, MD). Statistics were run using Prism 8.4.0, t-tests were used to compare all groups to saline treated MRL*-lpr* mice; *p≤ 0.05, **p ≤ 0.01, ***p ≤ 0.001.

**
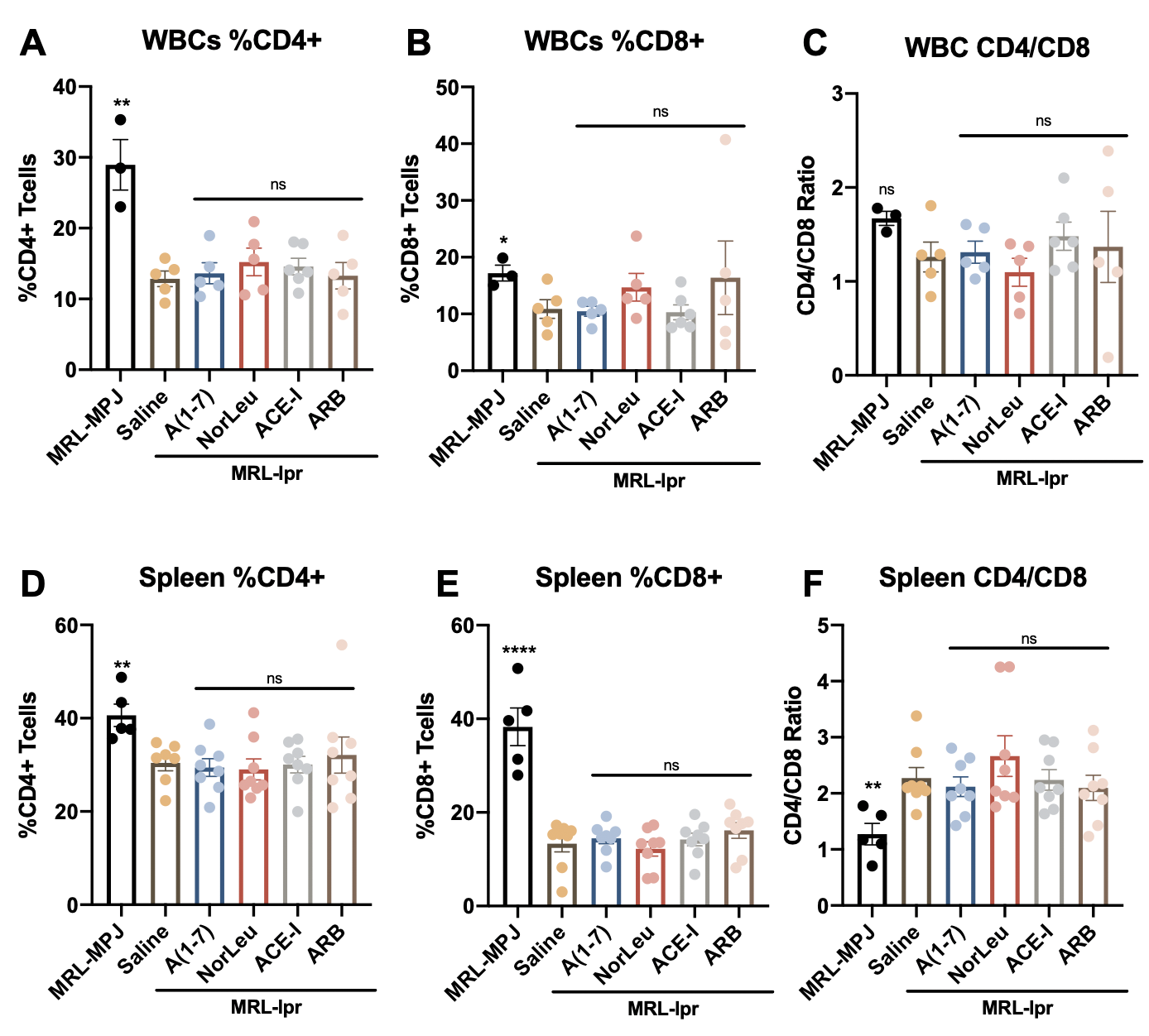
**

**Supplemental Figure 5. T cell composition in circulation and in the spleen is not affected by RAS modulation.** WBCs were and splenic cells were isolated from whole blood at necropsy and stained for T cell markers (CD3, CD4, CD8). Samples were then analyzed by flow cytometry. The % of CD4^+^ (A & D) and CD8^+^ (B & E) T cells in the blood (A & B) and spleen (D & E) were measured. The CD4/CD8 ratio was calculated for WBC (C) and splenic (F) samples. Statistics were run using Prism 8.4.0; t-tests were used to compare all groups; *p≤ 0.05, **p ≤ 0.01, ****p ≤ 0.0001.

**
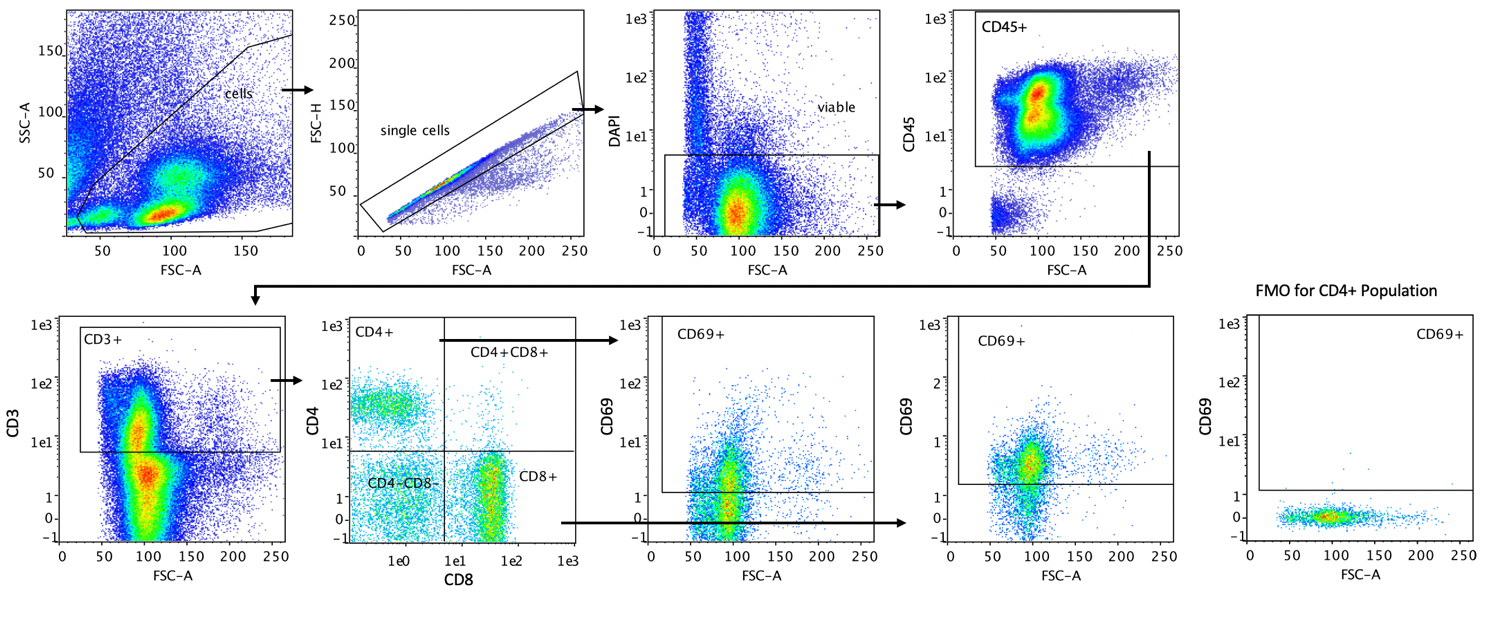
**

**Supplemental Figure 6. Gating strategy for the analysis of T cells and T cell activation in blood and spleen samples.** At necropsy, WBCs and splenic cells were isolated and stained for T cell markers (CD3, CD4, CD8), the T cell activation marker CD69. Single cell suspensions were then stained and analyzed by flow cytometry. DAPI was used to discriminate between live and dead cells. Immune cells were characterized as CD45^+^ and T cells as CD3^+^. T cells were further defined as CD4^+^ T cells or CD8^+^ T cells. Activated CD4^+^ T cells or CD8^+^ T cells were then characterized as CD69^+^.


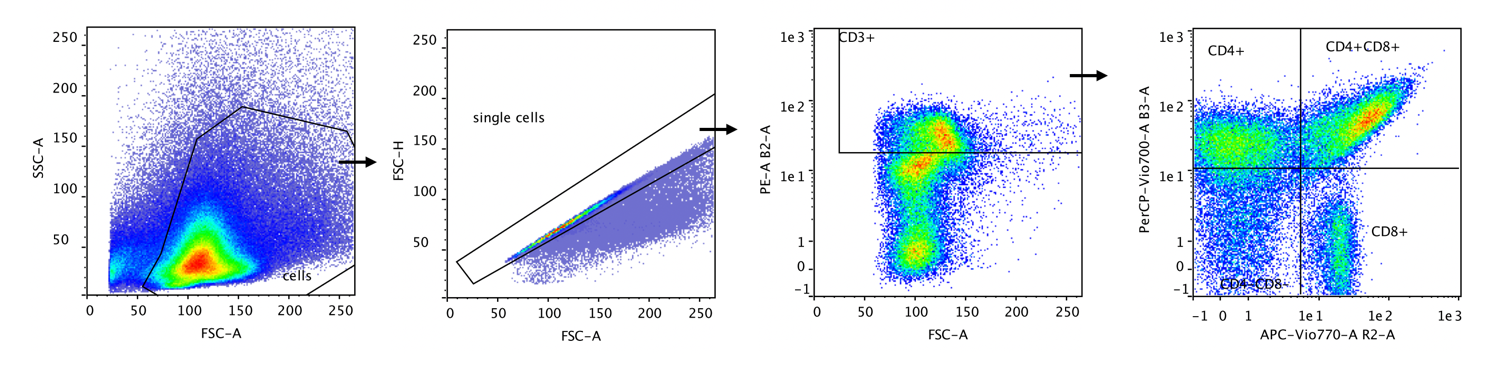


**Supplemental Figure 7. Gating strategy for the analysis of activated T cells spleen samples to look for IFN-**γ**, TNF-**α **and IL-10 producing cells.** At necropsy splenic cells were isolated, stimulated for 16hrs with PMA/ionomycin, and then stained with T cell markers (CD3, CD4 and CD8) and for IFN-γ, TNF-α, and IL-10 production. T cells were first characterized as CD3+ and further defined as CD4^+^ T cells or CD8^+^ T cells. Gates and results for IFN-γ and TNF-α producing cells are shown in Figure 4. Gates and results for IL-10 are shown in Figure 5.


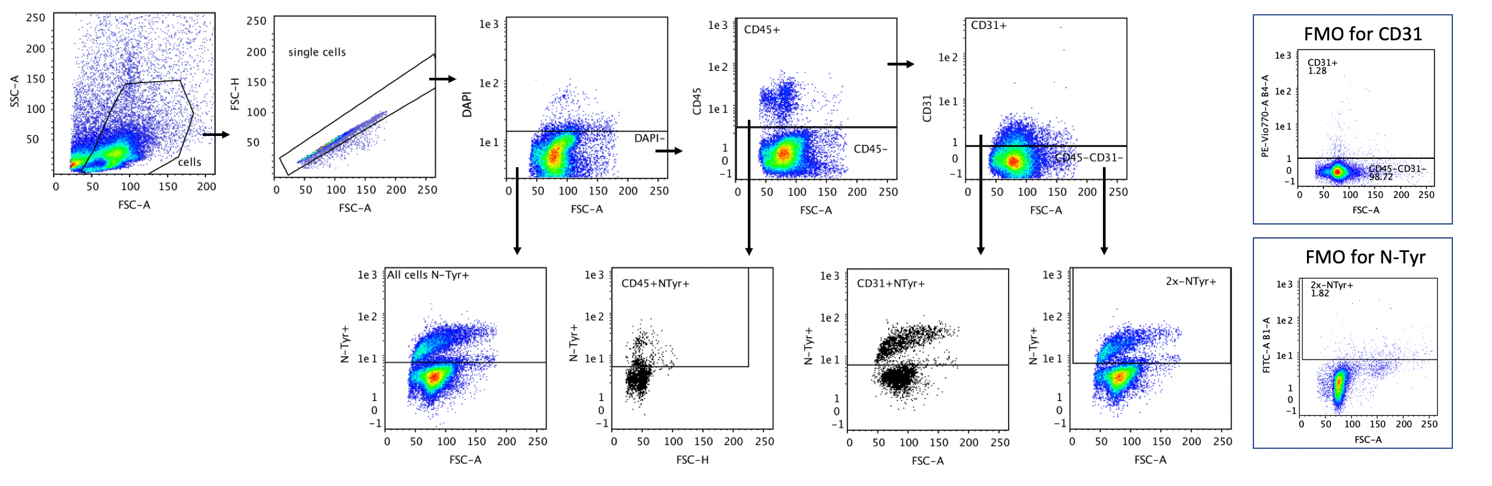


**Supplemental Figure 8. Gating strategy to measure N-tyr staining in cells from the kidney.**  The right kidney was collected at necropsy and dissociated into a single cell suspension, cells were then stained for immune and endothelial cell markers (CD45 & CD31), and N-tyr as a marker for OS. Stained cells were then analyzed by flow cytometry. DAPI was used to discriminate between live and dead cells. Immune cells were characterized as CD45^+^ and endothelial cells as CD31^+^. N-tyr was measured in all DAPI^-^ cells, CD45^+^ cells, CD31^+^ cells, and CD45^-^CD31^-^ cells which we presume to be kidney cells.
